# Selection and Validation of Reference Genes in Virus-Infected Sweet Potato Plants

**DOI:** 10.3390/genes14071477

**Published:** 2023-07-19

**Authors:** Guangyan Li, Xiaohui Sun, Xiaoping Zhu, Bin Wu, Hao Hong, Zhimei Xin, Xiangqi Xin, Jiejun Peng, Shanshan Jiang

**Affiliations:** 1 Shandong Key Laboratory of Plant Virology, Institute of Plant Protection, Shandong Academy of Agricultural Sciences, Jinan 250100, China; 2 Shandong Provincial Key Laboratory for Biology of Vegetable Diseases and Insect Pests, College of Plant Protection, Shandong Agricultural University, Taian 271018, China; 3 State Key Laboratory for Managing Biotic and Chemical Threats to the Quality and Safety of Agroproducts, Key Laboratory of Biotechnology in Plant Protection of MARA and Zhejiang Province, Institute of Plant Virology, Ningbo University, Ningbo 315211, China

**Keywords:** expression stability, quantitative real-time PCR, reference genes, experimental conditions

## Abstract

Quantitative real-time PCR (qRT-PCR) in sweet potatoes requires accurate data normalization; however, there are insufficient studies on appropriate reference genes for gene expression analysis. We examined variations in the expression of eight candidate reference genes in the leaf and root tissues of sweet potatoes (eight nonvirus-infected or eight virus-infected samples). Parallel analyses with geNorm, NormFinder, and Best-Keeper show that different viral infections and origin tissues affect the expression levels of these genes. Based on the results of the evaluation of the three software, the *adenosine diphosphate-ribosylation factor* is suitable for nonvirus or virus-infected sweet potato leaves. *Cyclophilin* and *ubiquitin* extension proteins are suitable for nonvirus-infected sweet potato leaves. *Phospholipase D1 alpha* is suitable for virus-infected sweet potato leaves. *Actin* is suitable for roots of nonvirus-infected sweet potatoes. *Glyceraldehyde-3-phosphate dehydrogenase* is suitable for virus-infected sweet potato roots. The research provides appropriate reference genes for further analysis in leaf and root samples of viruses in sweet potatoes.

## 1. Introduction

Quantitative real−time PCR (qRT−PCR) is a method that uses fluorescence to detect gene expression signals monitoring [1]. The method has high specificity and high sensitivity, is simple to operate, and is more easily operated than other molecular techniques [1]. However, the accuracy of this quantitative analysis depends on the number of initial templates, RNA quality, and enzyme reaction efficiency [2]. Therefore, in order to regulate the expression level of target genes, it is very important to find suitable internal reference genes to reduce the bias associated with qRT−PCR results [3].

The stable expression of internal reference genes in different organs and tissues is also known as family genes, and its products are essential for maintaining various basic activities of cells. In plants, reference genes mainly include *β*−*tubulin* (*TUB*), *18S ribosomal RNA* (*18S rRNA*), *actin* (*ACT*), etc. [4]. In general, internal reference genes must be expressed continuously and stably in different stages of growth and development, different tissues, and different stress conditions [5]. However, studies show that internal reference gene expression levels are unstable under different experimental conditions [6]. It is necessary to select the appropriate reference gene in the experiment.

At present, there are many studies on the selection of reference genes in plants [7,8,9]. Studies have shown that differences in the expression of common reference genes, such as *18S rRNA*, *glyceraldehyde*−*3*−*phosphate dehydrogenase* (*GAPDH*), and *ACT* under different experimental treatments are statistically significant [10,11,12]. Jain et al. (2006) found that the reference genes stably expressed in rice under different stress treatments were *18S rRNA* and *25S rRNA*, and the reference genes stably expressed in different periods and locations were *elongation factor 1*−*α* (*EF1−α*) and *ubiquitin 5* (*UBQ5*) [13]. Studies have shown that both biological and abiotic factors affect the expression of internal reference genes. When plant viruses infect the host, they can also affect host cell processes, and most of these processes involve factors encoded by housekeeping genes [14].

In plant virus studies, most studies involving gene expression are carried out on leaves, and most studies to identify and verify stable reference genes are also carried out on leaves: the transcripts of the *sand family protein*, *EF1−α*, *F-box family protein*, and *protodermal factor 2* genes in Arabidopsis leaves infected by five plant viruses were the most stable [15]. At the same time, some studies focused on differences between root and leaf reference gene transcripts. The analysis of tomato roots and leaves by BestKeeper, NormFinder, and GeNorm showed that different sources, different viruses, and different parts had an influence on the expression of reference genes [16]. *GAPDH* and *Ubiquitin (UBI)* were more stable in the root and leaf tissues of tomato, while *cyclophilin* was more stable in the root tissues [16]. Some studies have selected reference genes for stable transcription in fruits [8]. Different viral infections in a single host also affect the expression of reference genes [17].

Reference genes in sweet potatoes have also been reported before. Park et al. (2012) selected ten candidate reference genes for sweet potatoes to find stable reference genes under abiotic stress, and the results showed that the *adenosine diphosphate-ribosylation factor* (*ARF*)*, UBI, cyclooxygenase* (*COX*)*, GAPDH and ribosomal protein L* (*RPL*) genes were confirmed as reference gene sets applicable to all varieties of sweet potato [9]. However, whether virus infection can affect the stability of internal reference genes in sweet potatoes has not been reported, and whether stable internal reference genes under abiotic stress are affected by virus infection has not been verified. To study the interaction between the sweet potato virus and host genes and verify the function of host genes, a large number of quantitative analyses are needed. Therefore, it is necessary to screen the stable expression of internal reference genes in sweet potatoes under the condition of virus infection. The aim of this study was to screen the internal reference genes that were stably expressed in the roots and leaves of sweet potatoes that were uninfected or infected with a virus. Eight candidate reference genes, including *ACT*, *alpha tubulin* (*ATUB*), *cyclophilin* (*CYP*), *GAPDH*, *UBI*, *ARF*, *phospholipase D1 alpha* (*PLD*), and *18S rRNA*.

## 2. Materials and Methods

### 2.1. Plant Materials

In April 2019, samples showing typical symptoms of virus disease or asymptomatic sweet potato were collected from sweet potato seedbeds in Linyi City, Shandong Province, and planted separately in greenhouses covered with insect-proof net. The leaves and root tubers of eight virus−infected and eight nonvirus−infected sweet potato (Jishu25) samples were selected. The test was repeated three times to ensure the reliability of the results.

### 2.2. Total RNA Extraction

Total RNA is extracted from each tissue using the Flying Shark^®^ Plant RNA Isolation Kit (polysaccharides and polyphenolics-rich) (Nobelab Biotechnology Co., Ltd., Beijing, China). The concentration and purity of RNA were measured by spectrophotometer. RNA absorption ratios of 1.8–2.0 are obtained for subsequent analysis at OD260 nm/OD280 nm.

### 2.3. Synthesis of cDNA Synthesis

The first-strand cDNA was synthesized using the ReverTra Ace−a^TM^ first−strand cDNA synthesis kit (TOYOBO, Osaka, Japan). cDNA was synthesized using 1 mg total sweet potato at 42 °C for 20 min, followed by inactivation with ReverTra Ace-α™ in a volume of 2.0 mL at 99 °C for 5 min.

### 2.4. Candidate Reference Genes Primer Design

We selected eight possible reference genes in this study. These genes were obtained from the NCBI database using the sequences of the reported reference genes. Primer Premier 6.0 was used to design qRT-PCR primers. Primer parameters were as follows: GC content 45–55%, PCR product length 100–200 bp, melting temperature (Tm) 58–62 °C, primer length 18–25 bp (Table S1).

### 2.5. Determination of Standard Preparation and Determination of Gene Amplification Efficiency

The reference gene sequence was amplified and connected to pMD18-T (TaKaRa, Japan) for sequencing verification. The target recombinant plasmid was used as a quantitative standard. The standard product was diluted 10 times for 6 gradients, and then the qRT-PCR test was performed, respectively. The standard curve was established between the amplified Ct value and the target gene copy number. Meanwhile, the formula E value was used to calculate the efficiency of amplification. The melting curve is also monitored.

### 2.6. Quantitative Real-Time PCR (qRT-PCR)

We performed qRT-PCR in 96-well blocks with an Applied Biosystems^®^ QuantStudioTM 6 Flex real-time PCR system (Applied Biosystems, Foster City, CA, USA). The final reaction volume was 10 µL, and each reaction contained 2 × SYBR Green Supermix (Applied Biosystems, Foster City, CA, USA) 7.5 µL, 10 µM Forward Primer 0.45 µL, 10 µM Reverse Primer 0.45 µL, cDNA 1 µL, and DEPC H_2_O 0.6 µL. The amplification process included a denaturation step at 95 °C for 60 s, followed by 40 cycles of 95 °C for 15 s, 60 °C for 15 s, and 72 °C for 30 s. After cycling, qRT-PCR amplification melting curves were achieved by raising the temperature from 60 °C to 95 °C.

### 2.7. Data Processing and Analysis

GeNorm (version 3.5) [18], NormFinder (version 0.953) [19], and BestKeeper (version 1.0) [20] were used to identify stable reference genes. The stability of the reference gene depends on the relative expression of the gene in the sample. SPSS software was used for statistical analysis.

## 3. Results

### 3.1. Verification of Primer Specificity and Specificity Analysis

**Figure 1:**
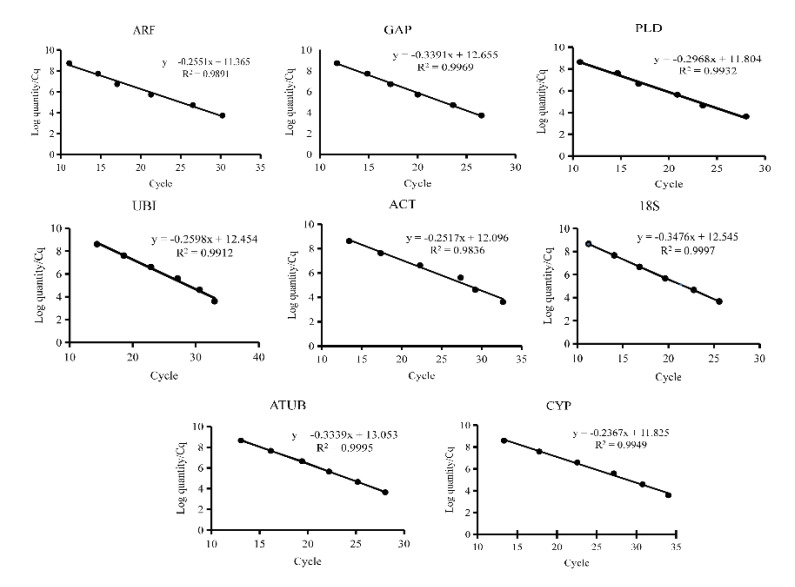
Quantitative real−time PCR standard curves of eight candidate reference genes. Eight candidate reference genes were shown as *adenosine diphosphate-ribosylation factor (ARF), glyceraldehyde*−*3*−*phosphate dehydrogenase (GAP), phospholipase D1 alpha (PLD), Ubiquitin (UBI), actin (ACT), 18S ribosomal RNA (18S), alpha tubulin (ATUB),* and *cyclophilin (CYP)*.

**Figure 2:**
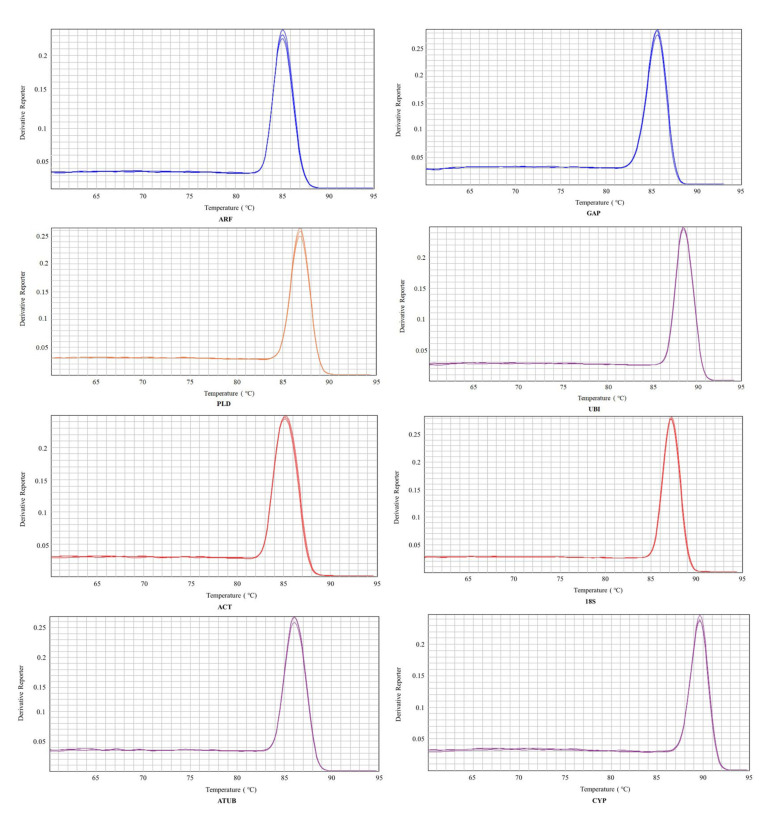
Melting curves of eight candidate reference genes. Eight candidate reference genes were shown as *adenosine diphosphate-ribosylation factor (ARF), glyceraldehyde*−*3*−*phosphate dehydrogenase (GAP), phospholipase D1 alpha (PLD), Ubiquitin (UBI), actin (ACT), 18S ribosomal RNA (18S), alpha tubulin (ATUB),* and *cyclophilin (CYP)*.

Eight commonly used internal reference genes were selected as candidate genes to select the reference genes stably expressed in virus-infected and non-virus-infected samples, which are *ARF* (JX177359), *GAP* (JX177362), *PLD* (JX177360), *UBI* (JX177358), *ACT* (EU250003), *18S* (HM053484), *ATUB* (AB572296), and *CYP* (CB330939). Agarose gel electrophoresis and melting curve analysis were performed to test the specificity of the primers. The results showed that the melt curve analysis of 8 candidate genes had a specific single peak, good primers amplification efficiency, and correlation coefficient, indicating that the primers could be effectively amplified and had good specificity, which could be used for subsequent analysis (Figure 1 and Figure 2).

### 3.2. Ct Analysis of Reference Genes in Leaves or Roots of Infected Sweet Potatoes

**Figure 3:**
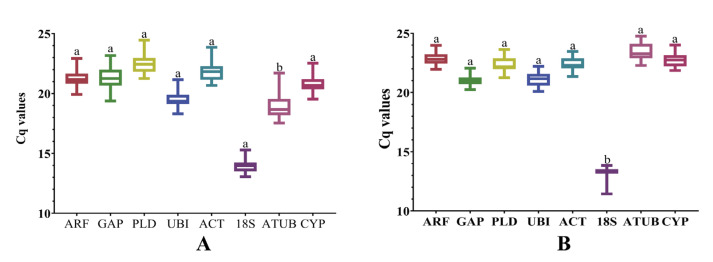
Graphic representation of expression data in relation to candidate reference genes. (**A**) Expression of candidate reference genes in susceptible leaves. (**B**) Expression of candidate reference genes at the pathogenetic root. Univariate analysis of variance was used to indicate statistical significance with different letters. Eight candidate reference genes were shown as *adenosine diphosphate-ribosylation factor (ARF), glyceraldehyde*−*3*−*phosphate dehydrogenase (GAP), phospholipase D1 alpha (PLD), Ubiquitin (UBI), actin (ACT), 18S ribosomal RNA (18S), alpha tubulin (ATUB),* and *cyclophilin (CYP)*.

Analysis of Ct values obtained from all biological and technical repeats of eight candidate reference genes from leaf samples, which were infected with viruses, showed that the general variation range of Ct values was 13 to 25, and the variation range of Ct values of each candidate reference gene was less than 5, indicating that the eight candidate genes could be used for the analysis of the reference gene in leaf samples (Figure 3A). Internal reference gene screening was performed on root tuber materials infected with viruses. In all samples, the Ct value of the eight candidate genes ranges from 12 to 25, and the change in the Ct value of each candidate reference gene is less than 5, which was relatively small, indicating that the eight candidate genes could be used for the analysis of susceptible root samples (Figure 3B).

### 3.3. Stability Analysis of Candidate Genes

To determine the most stable sweet potato reference genes, BestKeeper, NormFinder, and geNorm evaluated the stability of all candidate reference genes.

#### 3.3.1. GeNorm Analysis of Candidate Reference Genes in Leaves or Roots of Sweet Potato

**Figure 4:**
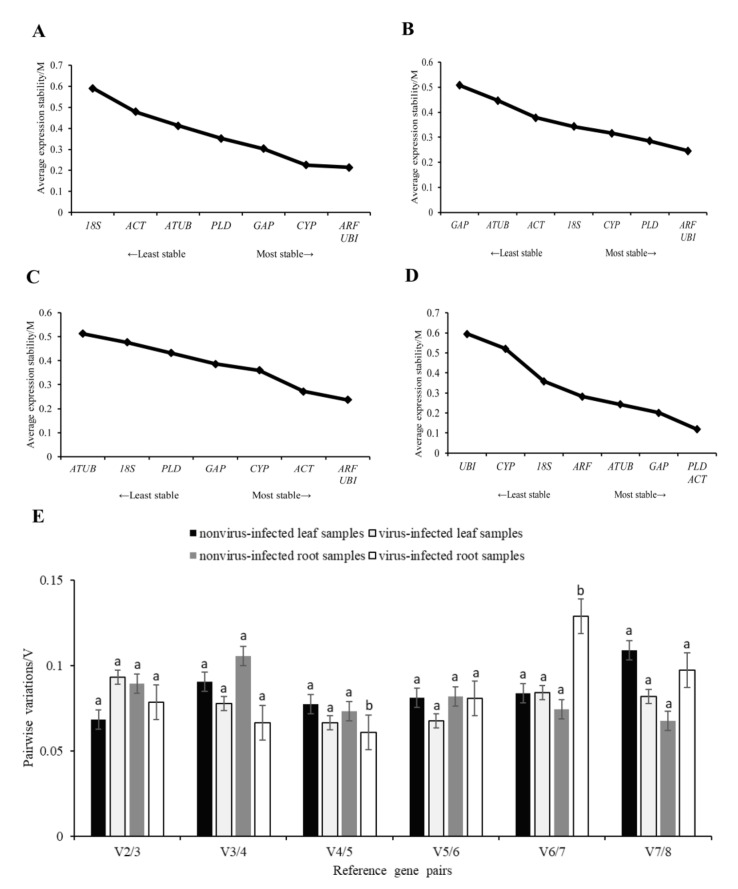
The stability of candidate reference genes, which are analyzed by geNorm. The average expression stability value (M) of eight candidate reference genes in the sample. (**A**) nonvirus−infected leaf sample, (**B**) virus−infected leaf sample, (**C**) nonvirus-infected root sample, (**D**) virus−infected root sample, (**E**) Determination of the optimal number of reference genes in accordance with the analysis of GeNorm. Variation in pairs V2/3, when the number of reference genes increases from two to three. Stepwise integration of less stable normalization factors generates the next data points (V3/4 to V7/8). A decrease in the V value indicates a positive effect of an additional gene for a reliable calculation of quantitative real-time PCR normalization. Univariate analysis of variance was used to indicate statistical significance with different letters. Eight candidate reference genes were shown as *adenosine diphosphate-ribosylation factor (ARF), glyceraldehyde*−*3*−*phosphate dehydrogenase (GAP), phospholipase D1 alpha (PLD), Ubiquitin (UBI), actin (ACT), 18S ribosomal RNA (18S), alpha tubulin (ATUB),* and *cyclophilin (CYP)*.

The M values of each gene were analyzed by geNorm software. The results showed that the M values of *ARF* and *UBI* were the lowest in the nonvirus-infected leaf samples, followed by *CYP* (Figure 4A). In leaf samples infected with a virus, the M values of *ARF* and *UBI* were the lowest, followed by *PLD* (Figure 4B), indicating that the relatively stable internal reference genes were *ARF*, *UBI*, and *CYP* in the nonvirus-infected samples, and *ARF*, *UBI*, and *PLD* in the virus-infected samples. The M values of *ARF* and *UBI* were the lowest in the nonvirus-infected root samples, followed by *ACT* (Figure 4C). In the virus-infected root samples, the M values of *PLD* and *ACT* were the lowest, followed by *GAP* (Figure 4D).

The V2/3 values of the paired variation analysis were less than 0.15, indicating that the selection of two internal reference genes for the analysis was sufficient and the introduction of a third gene was not necessary to eliminate the differences (Figure 4E). Overall analysis showed that the best candidate reference gene combination recommended by geNorm software was *ARF* and *UBI* in leaf and nonvirus-infected root samples. *PLD* and *ACT* were recommended as the best candidate reference gene combinations in virus-infected root samples.

#### 3.3.2. NormFinder Analysis of Candidate Reference Genes in Leaves or Roots of Sweet Potato

**Figure 5:**
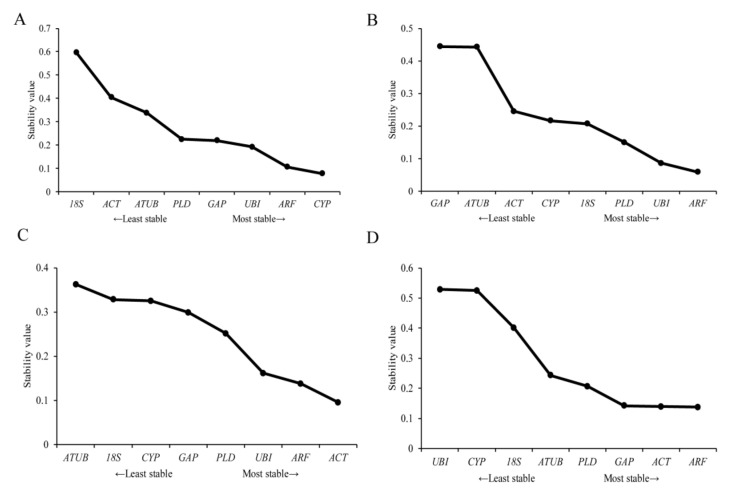
NormFinder analyses the expression stability of candidate reference genes. (**A**) nonvirus-infected leaf samples, (**B**) virus-infected leaf samples, (**C**) nonvirus-infected root samples, (**D**) virus-infected root samples. Eight candidate reference genes were shown as *adenosine diphosphate-ribosylation factor (ARF), glyceraldehyde*−*3*−*phosphate dehydrogenase (GAP), phospholipase D1 alpha (PLD), Ubiquitin (UBI), actin (ACT), 18S ribosomal RNA (18S), alpha tubulin (ATUB),* and *cyclophilin (CYP)*.

The M values of each reference gene were analyzed using NormFinder software. The results showed that the M values of *CYP* were the lowest in nonvirus-infected leaf samples, followed by *ARF* and *UBI* (Figure 5A). In leaf samples infected with a virus, the M value of ARF was the lowest, followed by *UBI* and *PLD* (Figure 5B). The results showed that the relatively stable internal reference genes were *ARF* and *UBI* in the leaves.

NormFinder software was used to analyze the M value of each gene. The results showed that the M value of *ACT* was the smallest in the nonvirus-infected root samples, followed by *ARF* and *UBI* (Figure 5C). In the virus-infected root samples, the M value of *ARF* was the smallest, followed by *ACT* and *GAP* (Figure 5D). The results showed that *ARF* and *ACT* are relatively stable internal reference genes in root samples.

#### 3.3.3. BestKeeper Analysis of Candidate Reference Genes in Leaves or Roots of Sweet Potato

The average Ct value of each leaf sample was analyzed using BestKeeper software. The standard deviation (SD) shows the stability of the eight candidate reference genes. In leaf samples without virus infection, BestKeeper highlighted *ATUB*, *18S*, *CYP*, *UBI*, *ARF*, *ACT,* and *GAP* in classification order, all characterized by the least overall variation, with SD [x-fold] < 2 and SD [Ct] < 1 (Table 1), which represents an acceptable expression change. In leaf samples infected with a virus, BestKeeper highlighted *CYP*, *ARF*, and *PLD*.

In root samples without viruses, BestKeeper highlighted *18S*, *GAP*, *CYP, and ACT* in rank order, all characterized by the lowest overall variation, with SD [x-fold] < 2 and SD [Ct] < 1 (Table 1). In virus-infected root samples, BestKeeper highlighted *CYP*, *PLD*, and *GAP*.

The evaluation results of the geNorm, NormFinder, and BestKeeper software in nonvirus-infected leaf samples, the most stable reference genes evaluated by the geNorm software were ARF, UBI, and CYP, while the most stable reference genes evaluated by the NormFinde software were CYP, ARF, and UBI. The stable reference genes selected by BestKeeper were ATUB, 18S, CYP, UBI, ARF, ACT, and GAP. Therefore, based on the evaluation results of the three software, the reference gene with the best stability in nonvirus-infected root samples is CYP, ARF, and UBI. In leaf samples infected with viruses, the most stable reference genes evaluated by geNorm and NormFinder software were ARF, UBI, and PLD. The stable reference genes selected by BestKeeper were CYP, ARF, and PLD. Therefore, based on the evaluation results of the three software, the reference gene with the best stability in nonvirus-infected root samples is ARF, PLD.

In nonvirus-infected root samples, the most stable reference genes evaluated by geNorm software were ARF, UBI, and ACT, while the most stable reference genes evaluated by NormFinde software were ACT, ARF, and UBI. The stable reference genes selected by BestKeeper were 18S, GAP, CYP, and ACT. Therefore, based on the evaluation results of the three software, the reference gene with the best stability in nonvirus-infected root samples is ACT. In virus-infected root samples, the most stable reference genes evaluated by geNorm software were PLD, ACT, and GAP, while the most stable reference genes evaluated by NormFinde software were ARF, ACT, and GAP. The stable reference genes selected by BestKeeper were CYP, PLD, and GAP. Therefore, based on the evaluation results of the three software, the reference gene with the best stability in nonvirus-infected root samples is GAP.

## 4. Discussion

qPCR is widely used to analyze gene expression because of its versatility and accuracy [21]. Normalization of target gene expression with reliable reference genes is important for the accuracy and reproducibility of data analysis [22]. However, qPCR requires stably expressed genes as reference genes to provide accurate results for gene expression analysis [13]. In plant research, the specific conditions of plants, species, and cultivars all affect the stability of reference genes, so the selection of appropriate internal reference genes must be determined by experiments. Currently, there are no reference genes that are stably expressed under all experimental conditions. Guenin et al. (2009) pointed out that reference genes must also be tested in different tissues of the same plant under different experimental conditions [23]. Therefore, in order to ensure the reliability and accuracy of qPCR data correction and normalization, stable internal reference genes should be selected under specific experimental conditions. In this study, eight internal reference genes were analyzed, and stability in nonvirus-infected or virus-infected sweet potato leaves and root samples was classified using three different statistical software: geNorm, NormFinder, and BestKeeper.

Sweet potato virus disease has become one of the main factors restricting the development of the sweet potato industry, and there are still many gaps in current research on the prevention and control technology of sweet potato virus disease and the control technology and the pathogenic mechanism of the virus [24]. There are many types of sweet potato viruses, and there are usually multiple viruses in the same sweet potato plant at the same time, and the co-infection of multiple sweet potato viruses will aggravate the damage of viral diseases [25]. The manifestations of the leaf disease of the sweet potato virus are complex and diverse, and samples infected with the same type of virus can also show different symptoms [26]. Viruses can also be detected in some asymptomatic samples, but the number and type of viruses detected are much lower than in symptomatic samples [27]. In general, internal reference gene expression can be stably expressed in various stages of the individual, in different tissues, or under different stress conditions [28]. However, a large number of studies have shown that no internal reference gene has met this condition fully and that the expression level of the main internal reference gene is currently related to organ type, stage, and external environmental conditions [9]. Therefore, one reference gene cannot be applied to all plants or different tissues and organs, and the best choice is that the expression of internal reference genes changes the least in the tissues and organs studied. Previous studies have shown that sweet potatoes are exposed to drought, salt, cold, and oxidative stress. The UBI, COX, ARF, GAP, and RPL genes were considered to be the most suitable reference genes in sweet potatoes. Interestingly, although the ACT and TUB genes are widely used, they are not the most suitable reference genes for different sets of sweet potato samples [9]. Yu et al. analyzed 16 commonly used reference genes in five different tissues under two different temperature stress conditions. Data analyses of Delta CT, geNorm, NormFinder, and BestKeeper have revealed that IbelF is the most stable gene and IbUBI the least stable gene as a reference [29]. EF1α and TUA were the most suitable reference genes in different tissues of sweet potato, eIF4α, and EF1α were the most stable reference genes under drought and low-temperature stress, and EF1α and ACT were the most stable reference genes under salt stress [30].

In this study, the results showed that the internal reference gene ARF is suitable for leaves without virus infection, which was consistent with the study that ARF was one of the most stable internal reference genes expressed in sweet potatoes after a variety of abiotic stress [9]. CYP and UBI are suitable for nonvirus-infected sweet potato leaves. PLD is suitable for virus-infected sweet potato leaves. ACT is suitable for roots of nonvirus-infected sweet potatoes. GAP is suitable for root-infected sweet potatoes. Previous studies have shown that, in most cases, all gene expression does not depend on abiotic stress or viral infection. Beta-tubulin (TUBB), GAPDH, and 18S rRNA are the three most stable genes. However, EF1A, EIF4A, and 28S rRNA for barley and oat samples and TUBA in wheat samples have consistently been rated as less reliable controls [31]. In tomatoes infected by different viruses, GAPDH and UBI were the most stable reference genes in leaf and root tissues; ACT and uridylate kinase were stably expressed throughout the tissue, while cyclophilin was stably expressed only in the root. 18S and EF1A are highly variable and do not apply to standardization [16]. Therefore, the expression of reference genes can be affected to a certain extent by variety differences, abiotic stress, or virus infection, and the selection of appropriate reference genes according to different plant states is of great significance to the accuracy of the results.

## 5. Conclusions

In summary, this study validates candidate reference genes from plant or root samples infected with a virus or nonvirus in sweet potatoes and performs a standardized gene expression analysis using qRT−PCR. Our results indicate that CYP and UBI are suitable for nonvirus-infected sweet potato leaves. PLD is suitable for the leaves of sweet potatoes infected with viruses. ACT is suitable for root crops of nonviral sweet potatoes. GAP is suitable for the root of sweet potatoes infected with viruses. Studies have shown that different appropriate reference genes should be chosen depending on specific experimental conditions. The screening results of this study provide technical support for standardized tests, later differential gene verification, and key gene extraction under conditions of sweet potato virus infection. It laid the foundation for a more accurate and extensive application of qRT−PCR in the analysis of sweet potato gene expression.

## Figures and Tables

**Table 1:** The BestKeeper period threshold was used to count eight candidate reference genes.

Classification		1	2	3	4	5	6	7	8
leaf samples not infected with viruses (*n* = 8)	Gene name	*ATUB*	*18S*	*CYP*	*UBI*	*ARF*	*ACT*	*GAP*	*PLD*
	coeff. of Corr. [r]	0.99	0.98	0.97	0.97	0.96	0.96	0.94	0.62
	coeff. of det. [r^2^]	0.97	0.96	0.95	0.95	0.93	0.92	0.88	0.39
	Geo Mean [Ct]	19.45	14.37	21.1	19.71	21.28	21.99	21.2	22.74
	min [Ct]	18.29	13.71	19.61	18.34	20.03	20.72	19.54	21.27
	max [Ct]	21.58	15.28	22.46	21.02	22.86	23.84	23.14	24.32
	SD [±Ct]	0.87	0.38	0.73	0.76	0.76	0.82	0.9	0.9
	CV [%]	4.46	2.61	3.48	3.85	3.57	3.72	4.25	3.97
	min [x-fold]	−2.23	−1.58	−2.81	−2.58	−2.38	−2.43	−3.15	−2.77
	max [x-fold]	4.39	1.87	2.58	2.49	2.98	3.59	3.82	2.98
	SD [±x-fold]	1.83	1.3	1.66	1.69	1.7	1.76	1.87	1.87
leaf samples infected with virus (*n* = 8)	Gene name	*CYP*	*ARF*	*PLD*	*18S*	*ACT*	*ATUB*	*GAP*	*UBI*
	coeff. of Corr. [r]	0.97	0.90	0.87	0.79	0.71	0.66	0.64	0.58
	coeff. of det. [r^2^]	0.94	0.81	0.75	0.63	0.51	0.44	0.4	0.34
	Geo Mean [Ct]	20.56	21.24	22.24	13.58	21.7	18.6	21.39	19.44
	min [Ct]	19.95	20.49	21.42	13.07	21.03	17.65	20.11	19.06
	max [Ct]	21.18	22.03	22.94	13.99	22.54	19.91	22.34	20.36
	SD [±Ct]	0.25	0.38	0.35	0.27	0.45	0.55	0.54	0.27
	CV [%]	1.22	1.77	1.59	2.01	2.08	2.93	2.52	1.37
	min [x-fold]	−1.52	−1.69	−1.76	−1.43	−1.59	−1.94	−2.43	−1.3
	max [x-fold]	1.54	1.73	1.62	1.32	1.78	2.48	1.94	1.89
	SD [±x-fold]	1.19	1.3	1.28	1.21	1.37	1.46	1.45	1.2
nonvirus-infected root samples (*n* = 8)	Gene name	*18S*	*GAP*	*CYP*	*ACT*	*ATUB*	*UBI*	*PLD*	*ARF*
	coeff. of Corr. [r]	0.99	0.95	0.94	0.81	0.78	0.75	0.72	0.47
	coeff. of det. [r^2^]	0.98	0.9	0.88	0.65	0.61	0.56	0.52	0.22
	Geo Mean [Ct]	13.41	21.1	22.78	22.73	23.92	21.16	22.79	23.04
	min [Ct]	13.08	20.24	21.97	22.03	22.44	20.09	22.01	21.97
	max [Ct]	13.84	22.05	24.01	23.47	24.77	22.21	23.64	23.98
	SD [±Ct]	0.2	0.45	0.5	0.43	0.53	0.48	0.43	0.51
	CV [%]	1.46	2.12	2.2	1.89	2.21	2.26	1.87	2.2
	min [x-fold]	−1.25	−1.82	−1.76	−1.62	−2.79	−2.1	−1.71	−2.1
	max [x-fold]	1.35	1.93	2.34	1.67	1.8	2.07	1.8	1.92
	SD [±x-fold]	1.15	1.36	1.42	1.35	1.44	1.39	1.34	1.42
virus-infected root samples (*n* = 8)	Gene name	*CYP*	*PLD*	*GAP*	*ACT*	*UBI*	*ARF*	*18S*	*ATUB*
	coeff. of Corr. [r]	0.95	0.91	0.84	0.62	0.58	0.27	−0.37	−0.39
	coeff. of det. [r^2^]	0.89	0.82	0.7	0.38	0.33	0.07	0.13	0.15
	Geo Mean [Ct]	22.64	21.96	20.9	22.09	21.02	22.61	13.02	23.01
	min [Ct]	21.86	21.26	20.47	21.35	20.14	22.35	11.44	22.28
	max [Ct]	23.52	22.7	21.27	22.62	21.79	22.91	13.73	23.73
	SD [±Ct]	0.52	0.3	0.26	0.26	0.52	0.17	0.41	0.31
	CV [%]	2.31	1.36	1.22	1.19	2.45	0.77	3.17	1.36
	min [x-fold]	−1.71	−1.63	−1.35	−1.67	−1.85	−1.2	−3	−1.65
	max [x-fold]	1.85	1.67	1.29	1.45	1.7	1.23	1.63	1.65
	SD [±x-fold]	1.44	1.23	1.19	1.2	1.43	1.13	1.33	1.24

Note. Eight candidate reference genes were shown as *adenosine diphosphate-ribosylation factor (ARF), glyceraldehyde-3-phosphate dehydrogenase (GAP), phospholipase D1 alpha (PLD), Ubiquitin (UBI), actin (ACT), 18S ribosomal RNA (18S), alpha tubulin (ATUB),* and *cyclophilin (CYP).*

## Data Availability

Not applicable.

## Supplementary Materials

The following supporting information can be downloaded at: https://www.mdpi.com/article/10.3390/xxxxx/s1, Table S1 Primer sequences for eight candidate reference genes.
