# Supplementary material for: Selection and Validation of Reference Genes in Virus-Infected Sweet Potato Plants
Source: Genes (Basel). 2023 Jul 19;14(7):1477. doi: 10.3390/genes14071477 (PMC10379385; doi:10.3390/genes14071477)
Supplement: Supplementary file 1 [file genes-14-01477-s001.zip › genes-2475310-supplementary.pdf]

Table S1. Primer sequences for eight candidate reference genes

Gene name	Gene symbol	GenBank Acc.	Primer sequence	Tm (° C)	Amplicon length (bp)
ADP-ribosylation factor	*ARF*	JX177359	For 5′- GGGATGCTGTGTTGCTTGTG-3′<br>Rev 5′-AGAGCCAGTCCAATCCCTCAT-3′	60	171
glyceraldehyde-3-phosphate dehydrogenase	*GAP*	JX177362	For 5′-ACTGTGCACGGACAATGGAAG-3′<br>Rev 5′-GCTTCAGCCCATGGAATCTC-3′	60	125
Phospholipase D1 alpha	*PLD*	JX177360	For 5′-TGATGGCGACGTAACAGAGCT-3′<br>Rev 5′-ATGAGGCAAGCAGTGTGGTG-3′	60	127
Ubiquitin extension protein	*UBI*	JX177358	For 5′-TCGCCGACTACAACATCCAG-3′<br>Rev 5′-TTCCTCAGCCTCTGCACCTTT-3′	60	189
Actin	*ACT*	EU250003	For 5′-CTTCGTGTTGCACCTGAGGA-3′<br>Rev 5′-CGTCCACTGGCATACAAGGAT-3′	60	161
18S ribosomal RNA	*18S*	HM053484	For 5′-ATGGTCGCAAGGCTGAAACTT-3′<br>Rev 5′-CAGACAAATCGCTCCACCAAC-3′	60	193
Alpha tubulin	*ATUB*	AB572296	For 5′-TCCAACTCCACCAGTGTTGC-3′<br>Rev 5′-TCTCCTTCCTCCATACCCTCA-3′	60	116
Cyclophilin	*CYP*	CB330939	For 5′-AACGGCTCGCAGTTCTTCATC-3′<br>Rev 5′-ACCGCCTCAGCCCTCTTAAT-3′	60	113
